# Genome Wide Phosphoproteome Analysis of *Zymomonas mobilis* Under Anaerobic, Aerobic, and N_2_-Fixing Conditions

**DOI:** 10.3389/fmicb.2019.01986

**Published:** 2019-09-04

**Authors:** Mehmet Tatli, Alexander S. Hebert, Joshua J. Coon, Daniel Amador-Noguez

**Affiliations:** ^1^DOE Great Lakes Bioenergy Research Center, University of Wisconsin-Madison, Madison, WI, United States; ^2^Department of Bacteriology, University of Wisconsin-Madison, Madison, WI, United States; ^3^Genome Center of Wisconsin, Madison, WI, United States; ^4^Department of Biomolecular Chemistry, University of Wisconsin-Madison, Madison, WI, United States; ^5^Department of Chemistry, University of Wisconsin-Madison, Madison, WI, United States; ^6^Morgridge Institute for Research, Madison, WI, United States

**Keywords:** *Z. mobilis*, protein phosphorylation, glycolysis, nitrogen fixation, ammonia assimilation, phosphoproteome

## Abstract

Protein phosphorylation is a post-translational modification with widespread regulatory roles in both eukaryotes and prokaryotes. Using mass spectrometry, we performed a genome wide investigation of protein phosphorylation in the non-model organism and biofuel producer *Zymomonas mobilis* under anaerobic, aerobic, and N_2_-fixing conditions. Our phosphoproteome analysis revealed 125 unique phosphorylated proteins, belonging to major pathways such as glycolysis, TCA cycle, electron transport, nitrogen metabolism, and protein synthesis. Quantitative analysis revealed significant and widespread changes in protein phosphorylation across growth conditions. For example, we observed increased phosphorylation of nearly all glycolytic enzymes and a large fraction of ribosomal proteins during aerobic and N_2_-fixing conditions. We also observed substantial changes in the phosphorylation status of enzymes and regulatory proteins involved in nitrogen fixation and ammonia assimilation during N_2_-fixing conditions, including nitrogenase, the Rnf electron transport complex, the transcription factor NifA, GS-GOGAT cycle enzymes, and the P_*II*_ regulatory protein. This suggested that protein phosphorylation may play an important role at regulating all aspects of nitrogen metabolism in *Z. mobilis*. This study provides new knowledge regarding the specific pathways and cellular processes that may be regulated by protein phosphorylation in this important industrial organism and provides a useful road map for future experiments that investigate the physiological role of specific phosphorylation events in *Z. mobilis*.

## Introduction

*Zymomonas mobilis*, a facultatively anaerobic alphaproteobacterium, possesses several desirable characteristics for industrial biofuel production. These include fast glucose catabolism, high ethanol yield (up to 96% of consumed glucose), low biomass production, resilience to inhibitors present in lignocellulosic hydrolysates, and tolerance to high ethanol and sugar concentrations (Rogers et al., 1980; Panesar et al., 2006; Pan et al., 2014; Yang et al., 2016a; Martien et al., 2019). In addition, *Z. mobilis* possesses nitrogenase and can efficiently fix atmospheric nitrogen (N_2_) without affecting ethanol yield, which adds potential economic and environmental benefits to ethanol production (Kremer et al., 2015). However, regulation of nitrogen fixation and metabolism in *Z. mobilis* remains largely unexplored (Jack et al., 2001; Huergo et al., 2013).

Unlike most anaerobic biofuel producers, *Z. mobilis* is highly tolerant to oxygen, although its growth rate and ethanol yield decrease significantly under aerobic conditions (Tanaka et al., 1990; Yang et al., 2009; Martien et al., 2019). Recent studies have indicated that during aerobic growth, oxidative damage to iron-sulfur (FeS) clusters constitutes a major factor influencing *Z. mobilis* metabolism and that respiratory enzymes and the ability to form multicellular aggregates are important for its survival (Jones-Burrage et al., 2019; Martien et al., 2019). Despite these and other recent advances (Yang et al., 2009; Rutkis et al., 2016; Strazdina et al., 2018), much remains to be learned about the regulation of *Z. mobilis* physiology during aerobic growth.

There is growing interest in redirecting *Z. mobilis* highly catabolic metabolism toward the production of advanced biofuels such as higher alcohols (e.g., isobutanol) and isoprenoid fuels derived from the methylerythritol phosphate (MEP) pathway (Yang et al., 2016b; Agrawal et al., 2017; Martien et al., 2019). However, a deeper understanding of the control and regulation of *Z. mobilis* metabolism will be required to achieve this.

Protein phosphorylation is best known for its widespread regulatory role in eukaryotes. However, a growing body of research now demonstrates that this post-translational modification is highly prevalent in bacteria, with potentially critical regulatory functions (Macek and Mijakovic, 2011; Soufi et al., 2012; Pisithkul et al., 2015; Ravikumar et al., 2015; Soares and Blackburn, 2016). Propelled by recent advances in mass spectrometry techniques, genome-wide phosphoproteome analyses have been reported in several industrially and medically relevant bacteria, including *Escherichia coli*, *Bacillus subtilis*, *Klebsiella pneumoniae*, *Streptomyces coelicolor, Rhodopseudomonas palustris* (Soung et al., 2009; Parker et al., 2010; Manteca et al., 2011; Lin et al., 2015), and others (Soufi et al., 2008; Aivaliotis et al., 2009; Ravichandran et al., 2009). These studies have identified large numbers of phosphorylation events on Ser, Thr, and Tyr residues, many of them in metabolic enzymes. Although, the regulatory functions of protein phosphorylation in bacteria are still largely undefined, these phosphoproteomics studies have provided the necessary foundation to investigate the physiological function of specific phosphorylation events in these bacterial species (Macek et al., 2007b; Soufi et al., 2008; Aivaliotis et al., 2009; Manteca et al., 2011; Ravikumar et al., 2014).

Here, using mass spectrometry, we report the first genome wide investigation of protein phosphorylation in the biofuel producer *Z. mobilis* across three different growth conditions: anaerobic growth, aerobic growth, and N_2_-fixing conditions. We identified 125 unique phosphorylated proteins distributed among primary metabolic pathways and cellular processes such as glycolysis, TCA cycle, protein biosynthesis, electron transport, and nitrogen fixation. Our analysis also revealed widespread changes in protein phosphorylation across these three growth conditions, providing novel insights regarding the specific pathways and cellular processes that may be regulated by protein phosphorylation in this important industrial organism.

## Materials and Methods

### Strain and Growth Conditions

*Zymomonas mobilis* ZM4 (ATCC 31821) strain was first grown on a rich medium plate anaerobically at 30°C for 3 days. This was followed by inoculation of rich medium with a single colony from the plate. A 2 mL ZM4 minimal medium was inoculated with liquid rich medium overnight culture and grown for 16 h. This culture was then used to inoculate a 25 mL minimal medium culture to a starting optical density of 0.05 (measured at 600 nm). The cells were grown to O.D_600_ of 0.5 before being collected by centrifugation for 15 min at 4500 rpm (Allegra X-30R, Beckman Coulter) (Supplementary Figure S1); cell pellets were stored at −80°C. Anaerobic growth experiments were performed in an anaerobic glove bag with an atmosphere of 5% H_2_ and 5% CO_2_, and 90% N_2_; oxygen level was kept < 50 ppm. For aerobic growth experiments, all steps were the same as anaerobic conditions with the exception that cultures were moved to ambient atmosphere at O.D_600_ of 0.2 and allowed to grow by stirring at 30°C until the cells reached to O.D_600_ of 0.5 at which point they were collected by spinning down at 4500 rpm for 15 min. For N_2_-fixing conditions, all steps used were the same as the anaerobic conditions, but 1 g/L NH_4_SO_4_ was omitted from the minimal medium. Rich media plates were prepared using 10 g/L yeast extract, 2 g/L KH_2_PO_4_, 18 g/L agar, and 20 g/L glucose. Minimal medium contained 1 g/L K_2_HPO_4_, 1 g/L KH_2_PO_4_, 0.5 g/L NaCl, 1 g/L NH_4_SO_4_, 0.2 g/L MgSO_4_ 7H_2_O, 25 mg/L Na_2_MoO_4_ 2H_2_O, 2.5 mg/L FeSO_4_ 7H_2_O, 20 mg/L CaCl_2_ 2H_2_O, 1 mg/L Calcium Pantothenate, and 20 g/L glucose.

### Lysis and Digestion

Lysis and digestion were performed as previously described (Ghosh et al., 2019). Briefly, the cell pellets were first re-suspended in 1 mL 6 M guanidine hydrochloride (GnHCl) to lyse the cells and precipitate the protein, MeOH was added to 90% final concentration. The samples were then centrifuged at 3,000 g for 15 min. The supernatant was discarded, and pellets were allowed to dry for approximately 5 min. The protein pellets were re-suspended in 1 mL 8 M urea, 100 mM Tris pH 8.0, 10 mM TCEP, and 40 mM chloroacetamide followed by dilution to 2 M urea using 50 mM Tris pH 8. Trypsin was added at approximate 100:1 ratio, and the samples were incubated overnight at ambient temperature. Trypsin was added again at 100:1 ratio, and the samples were incubated at ambient temperature for 1 h. Each sample was acidified with trifluoroacetic acid (TFA) and pelleted. The supernatant was desalted over a PS-DVB cartridge and dried down. The final peptide yield was estimated by absorbance at 205 nm using a nanodrop system (extinction coefficient = 31).

### Phosphorylation Enrichment

Phosphopeptide enrichment was performed using a previously described titanium (IV) immobilized metal affinity chromatography (Ti-MAC) method (Hebert et al., 2018). In brief, for each sample, 1 mg of peptides was combined with 100 μL of Ti-IMAC beads (ResynBio) in 6% TFA/80% ACN, washed three times with this buffer, then washed twice with 80% ACN, once with 0.5 M glycolic acid/80% ACN, and lastly washed twice in 80% ACN. Phosphopeptides were eluted with 50% ACN/1% ammonium hydroxide. The eluate was dried and then desalted over a PS-DVB cartridge.

### LC-MS/MS

For each phosphorylation analysis, 25% of the sample was loaded onto a 75 μm i.d. 30 cm long capillary with an imbedded electrospray emitter and packed with 1.7 μm C18 BEH stationary phase. Peptides were eluted with a gradient of acetonitrile over 100 min (Mobile phase A: 0.2% formic acid, Mobile phase B: 70% ACN with 0.2% formic acid as previously described (Hebert et al., 2014). Eluting peptides were analyzed with an Orbitrap Fusion Lumos. Survey scans were performed with the Orbitrap at 60,000 resolution. Data dependent top speed (1 s cycle time) MS/MS sampling of peptide precursors, with charge states +2 to +4, was performed with dynamic exclusion set to 30 s. The MS/MS sampling was performed with quadrupole isolation = 1.6 *m/z*, fragmentation by higher-energy collisional dissociation (HCD) with normalized collision energy (NCE) = 30, maximum injection time = 118 ms, AGC = 2 × 10^5^ and fragment ions were analyzed by the Orbitrap with R (resolution) = 60,000. Each sample was analyzed with and without the advanced precursor determination (APD) toggled (Hebert et al., 2018).

### Software and Data Analysis

Raw files were analyzed with MaxQuant software program; default settings were applied except for match between runs and label free quantitation, which were toggled on (Cox and Mann, 2008). The intensity values for each phosphosite were averaged across the replicate analyses for each sample. Phosphosites that were not localized or quantified in all bioreplicates of at least one condition were excluded from the quantitative analysis. A phosphosite was considered quantified if an MS1 signal for the peptide precursor was observed in back to back scans. This data was then processed with Perseus by first performing a log2 transformation, followed by missing value imputation using the default parameters (Tyanova et al., 2016). Student’s *T*-tests were performed across aerobic vs. anaerobic growth and N_2_-fixing conditions vs. anaerobic growth). These *p*-values were converted to *q*-values using permutation-based FDR correction. For normalization to protein abundance, phosphorylation log2 signals were mean normalized across each sample for each site independently, and the process was repeated for protein log2 label-free quantitation signals. A protein normalized signal was then calculated for each sample by subtracting the mean normalized protein value from the corresponding mean normalized phosphorylation site value, followed by calculating the fold changes and *q*-values in Perseus software platform.

## Results

### Analysis of *Zymomonas mobilis* Phosphoproteome

**FIGURE 1:**
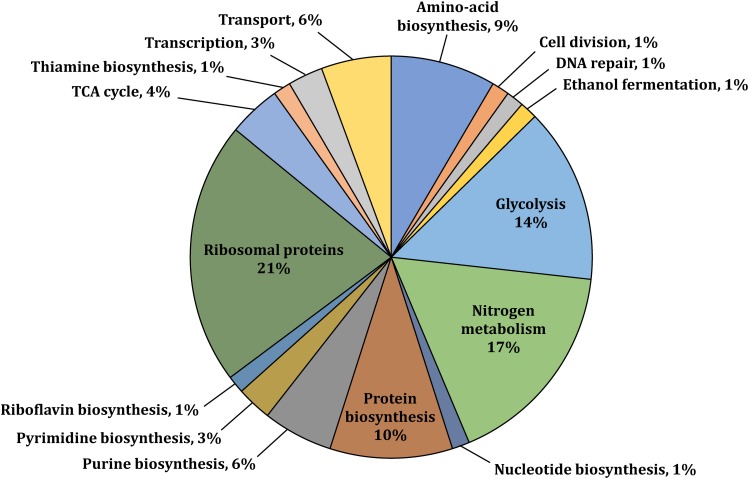
Functional classification of selected phosphoproteins with available gene annotation in UniProt or the KEGG Pathway databases (UniProt Consortium, 2008; Kanehisa et al., 2016). Phosphoproteins were identified in at least one of the three studied growth conditions (i.e., aerobic, anaerobic, or N_2_-fixing conditions).

We performed a genome wide investigation of protein phosphorylation in *Z. mobilis* across three different growth conditions: anaerobic, aerobic, and N_2_-fixing conditions. Cells were grown in defined minimal media using glucose as the single carbon source and ammonia as the nitrogen source, except for N_2_-fixing conditions in which no ammonia was added. For each condition, five biological replicates were generated. Samples for phosphoproteome analyses were taken during mid-exponential growth (i.e., OD_600_ ∼0.5) (Supplementary Figure S1). Tryptic peptides generated from these samples were enriched for phosphopeptides using a Ti(IV)-IMAC protocol described previously (Riley and Coon, 2016). Enriched phosphopeptides were analyzed by nano LC-MS/MS on an Orbitrap Fusion Lumos instrument and the resulting identifications were quantitatively compared by label free quantitation. In total, we identified 363 phosphorylation sites (Supplementary Table S1), of which 226 were confidently localized (≥75% probability of single residue localization) and 197 were both localized and quantified. To be considered quantified, the phosphopeptide must have a measurable MS1 signal (see section “Materials and Methods”) in all five biological replicates of at least one growth condition. The identified sites were distributed across 125 unique phosphorylated proteins (Supplementary Table S2). In parallel to phosphoproteome analyses, we performed comprehensive quantitative proteomics analyses to evaluate if changes in phosphorylation across growth conditions were stoichiometric (Supplementary Table S3). The number of phosphorylated proteins identified comprised 6.6% of all *Z. mobilis* proteins (125/1892 proteins). This fraction is similar to the fraction of phosphorylated proteins previously reported in other bacteria such as *E. coli* (9.1%) *B. subtilis* (4.2%), and *K. pneumoniae* (5.4%) (Table 1; Lin et al., 2015). We categorized the phosphoproteins that we identified according to their biological functions and found them to be distributed across essential cellular and metabolic processes such as glycolysis, TCA cycle, amino acid, nucleotide, and protein biosynthesis, nitrogen fixation, and ammonia assimilation (Figure 1). The serine/threonine/tyrosine (STY) distribution of phosphorylation sites across all phosphoproteins was 73% serine, 21% threonine, and 6% tyrosine (Table 1). This was very similar to the STY distributions found in *E. coli*, *B. subtilis*, and *K. pneumoniae* (Ravikumar et al., 2014; Lin et al., 2015; Potel et al., 2018), but somewhat different than *R. palustris* (STY: 63.5/16/19.5) and *S. coelicolor* (STY: 46.8/48/5.2) (Manteca et al., 2011; Hu et al., 2012; Table 1). Similarly to reports from other bacteria, we observed an enrichment of N-terminal phosphorylation sites in *Z. mobilis*: 15% of all phosphoproteins were phosphorylated on Ser^2^ or Thr^2^ (Supplementary Table S4). Interestingly, this fraction is higher than *E. coli* (6.12%), *B. subtilis* (6.71%), and *K. pneumoniae* (4.64%). We also observed that nearly all (>95%) proteins phosphorylated on Ser^2^ and Thr^2^ had their first methionine removed, as it has been observed in other bacteria (Lin et al., 2015; Supplementary Table S4).

**TABLE 1:** STY distribution of phosphorylation sites across all phosphoproteins.

	**Number of Genes**	**Number of Phosphoproteins**	**Number of Phosphopeptides**	**Number of Phosphosites**	**pS (%)**	**pT (%)**	**pY (%)**	**% of Phosphoproteins in genome**
**Gram positive bacteria**								
*B. subtilis*^a^	4188	175	441	226	74.8	17.7	7.1	4.2
*S. coelicolor*^b^	7825	127	260	289	46.8	48	5.2	1.6
**Gram negative bacteria**								
*Z. mobilis*^Δ^	1892	125	172	177	73	21	6	6.6
*E. coli*^a^	4316	392	1212	766	69.5	21.8	7.7	9.1
*K. pneumoniae*^a^	5262	286	663	388	72.9	13.7	12.9	5.4

*^*a*^Lin et al., 2015. ^*b*^Manteca et al., 2011. ^Δ^ This study.*

## Differential Phosphorylation Across Growth Conditions

**FIGURE 2:**
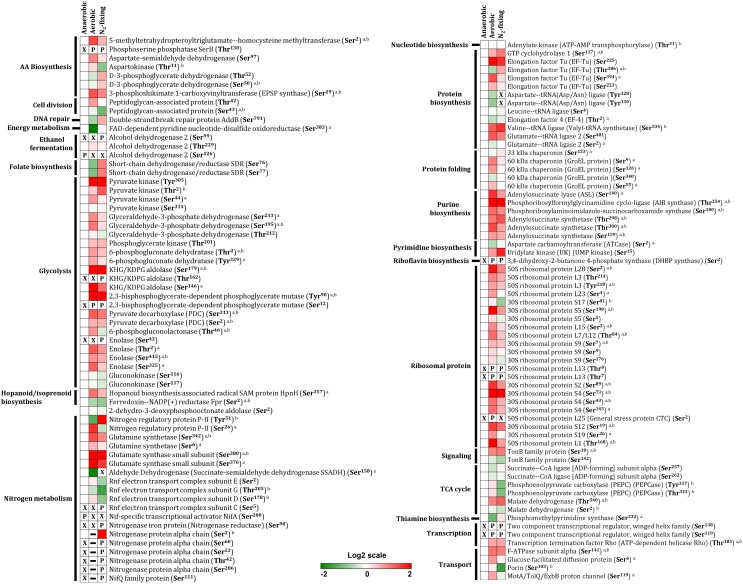
Differential protein phosphorylation across aerobic, anaerobic and N_2_-fixing growth conditions. The heatmap displays data for 79 selected phosphoproteins. Multiple phosphorylation sites were identified for several proteins. The red (high) and green (low) color scale indicates relative changes (log_2_ fold-changes vs. anaerobic samples) in protein phosphorylation across growth conditions. Data represents the average of 5 biological replicates. For proteins that were not phosphorylated in the anaerobic control samples and in at least one more growth condition, fold-change comparisons were not made; instead, changes in phosphorylation status are shown as follows: *X* indicates no phosphorylation was detected, *P* indicates site is phosphorylated, and a dash (–) indicates that protein level was not quantitated due to its low abundance in the specified growth condition and phosphorylation was also not detected. For fold-change comparisons, the superscripts *a* and *b* indicate significant (adjusted *q* < 0.05, see section “Materials and Methods”) changes in phosphorylation under aerobic and N_2_-fixing conditions, respectively.

Our analysis revealed widespread changes in protein phosphorylation across anaerobic, aerobic, and N_2_-fixing growth conditions that affected several major pathways and cellular processes (Figure 2). Below we summarize a subset of the most significant alterations.

### Glycolysis and TCA Cycle

**FIGURE 3:**
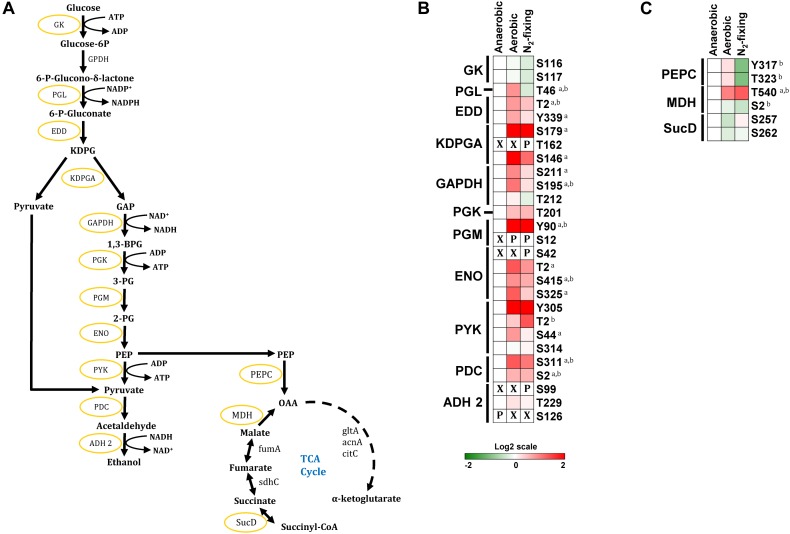
Phosphorylation of central carbon metabolic enzymes. **(A)** The Entner–Doudoroff glycolytic pathway, TCA cycle, and ethanol fermentation pathway in *Z. mobilis*. Yellow circles indicate phosphorylated proteins identified in this study. **(B,C)** Heatmaps showing differential phosphorylation of glycolytic, ethanol fermentation, and TCA cycle enzymes across growth conditions. The red (high) and green (low) color scale indicates relative changes (log_2_ fold-changes vs. anaerobic samples) in protein phosphorylation across growth conditions. Data represents the average of 5 biological replicates. For proteins that were not phosphorylated in the anaerobic control samples and in at least one more growth condition, fold-change comparisons were not made; instead, changes in phosphorylation status are shown as follows: *X* indicates no phosphorylation was detected, *P* indicates site is phosphorylated. For fold-change comparisons, the superscripts *a* and *b* indicate significant (adjusted *q* < 0.05, see section “Materials and Methods”) changes in phosphorylation under aerobic and N_2_-fixing conditions, respectively. Abbreviations: GK, Glucokinase; GPDH, Glucose-6-phosphate dehydrogenase; PGL, Phosphogluconolactonase; EDD, 6-phosphogluconate dehydratase; KDPGA, 2-keto-3-deoxy-6-phosphogluconate aldolase; GAPDH, Glyceraldehyde-3-phosphate dehydrogenase; PGK, Phosphoglycerate kinase; PGM, phosphoglycerate mutase; ENO, Enolase; PYK, Pyruvate kinase; PDC, Pyruvate decarboxylase; ADH 2, Alcohol dehydrogenase 2; PEPC, Phosphoenolpyruvate carboxylase; MDH, Malate dehydrogenase; fumA, Fumarate hydratase; SucD, Succinate–CoA ligase [ADP-forming] subunit alpha; gltA, Citrate synthase; acnA, Aconitate hydratase; citC, Isocitrate dehydrogenases; sdhC, Succinate dehydrogenase.

*Zymomonas mobilis* catabolizes sugars via the Entner–Doudoroff (ED) pathway (Figure 3A) instead of the well-known Embden–Meyerhof–Parnas (EMP) pathway found in model organisms such as *E. coli* and *Saccharomyces cerevisiae* (Sprenger, 1996; Inoshima et al., 2012). The ED and EMP pathways share a common set of reactions in lower glycolysis that convert glyceraldehyde-3-phosphate (GAP) into pyruvate but each pathway has unique reactions in its initial steps. The ED pathway starts with phosphorylation of glucose to generate glucose-6-phosphate (G6P), which is then oxidized and dehydrated to 2-keto-3-deoxy-6-phosphogluconate (KDPG). KDPG is then cleaved by KDPG aldolase (KDPGA, ZMO0997) into pyruvate and GAP. *Z. mobilis* converts >95% of the pyruvate produced from glucose into ethanol via pyruvate decarboxylase (PDC, ZMO1360) and alcohol dehydrogenase (ADH 1, ZMO1236; ADH 2, ZMO1596) (Figure 3A; Panesar et al., 2006).

We found that with the exception of glucose-6-phosphate dehydrogenase (GPDH, ZMO0367), all other enzymes in the ED glycolytic pathway were phosphorylated under at least one growth condition (Figure 3). We identified multiple phosphorylation sites in most enzymes in glycolysis (Figure 3B). For example, 6-phosphogluconate dehydratase (EDD, ZMO0368) had two phosphorylation sites (Thr^2^, Tyr^339^), glyceraldehyde phosphate dehydrogenase (GAPDH, ZMO0177) had three phosphorylation sites (Thr^212^, Ser^211^ and Ser^195^), and pyruvate kinase (PYK, ZMO0152) had four phosphorylation sites (Tyr^305^, Thr^2^, Ser^44^, Ser^314^). Interestingly, we observed a general trend for increased phosphorylation in nearly all glycolytic enzymes during aerobic and N_2_-fixing growth (Supplementary Table S4). Some phosphorylation sites were found only in specific growth conditions. For example, KDPGA and Enolase (ENO, ZMO1608) were phosphorylated at Thr^162^ and Ser^42^, respectively, only under N_2_-fixing conditions; while phosphoglycerate mutase (PGM, ZMO1240) was phosphorylated at Ser^12^ under aerobic and N_2_-fixing conditions but not under anaerobic growth. The high prevalence of phosphorylation sites in *Z. mobilis* glycolytic enzymes bears similarity to previous phosphoproteome analyses of other bacteria (i.e., *S. coelicolor*, *Lactococcus lactis*, *E. coli*, *B. subtilis*, and *K. pneumoniae*) showing that most of their glycolytic enzymes are phosphorylated (Macek et al., 2007a, b; Soufi et al., 2008; Manteca et al., 2011; Lin et al., 2015).

Within the ethanol fermentation pathway, we found multiple phosphorylation sites in PDC (Ser^2^ and Ser^311^) and ADH 2 (Ser^99^, Thr^229^, and, Ser^126^). PDC displayed significantly increased phosphorylation on both Ser^2^ and Ser^311^ under aerobic and N_2_-fixing conditions. In contrast, ADH 2 was specifically phosphorylated at Ser^126^ only under anaerobic growth and at Ser^99^ only under N_2_-fixing conditions (Figure 3B). Previous reports showed that ethanol yield is reduced to <40% under aerobic growth while it stayed largely unaffected under N_2_-fixing conditions (Kremer et al., 2015; Martien et al., 2019); thus, ADH 2 phosphorylation at Ser^99^ and Ser^126^ might play a regulatory role on ethanol biosynthesis in *Z. mobilis*.

In contrast to glycolytic enzymes, only a few enzymes associated with the TCA cycle were phosphorylated. Phosphoenolpyruvate carboxylase (PEPC, ZMO1496) was phosphorylated at Tyr^317^ and Thr^323^; succinate-CoA ligase (SucD, ZMO0567) was phosphorylated at Ser^257^ and Ser^262^, and putative malate dehydrogenase (MDH, ZMO1955) was phosphorylated at Ser^2^ and Thr^540^ (Figure 3C). Unlike glycolytic enzymes, we did not observe a generalized increase in phosphorylation of TCA cycle-related enzymes under aerobic and N_2_-fixing conditions (Figure 3C). Only MDH phosphorylation at Thr^540^ increased significantly under both aerobic and N_2_-fixing conditions while phosphorylation of PEPC (Tyr^317^ and Thr^323^) decreased significantly under N_2_-fixing conditions (Figure 3C).

### Nitrogen Fixation

**FIGURE 4:**
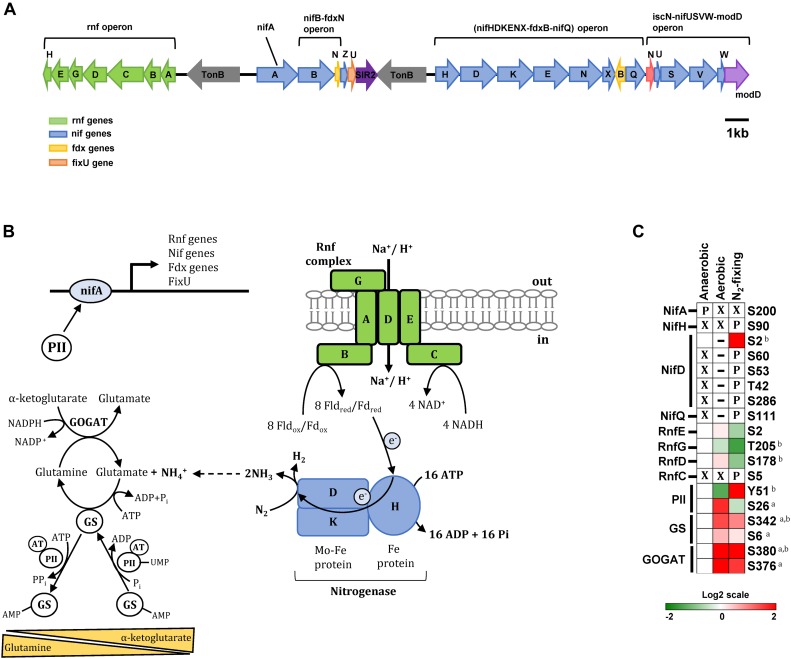
Phosphorylation of enzymes and regulatory proteins involved in nitrogen fixation and ammonia assimilation. **(A)** Nitrogen fixation genes are located within a single chromosomal region that includes the master regulator NifA, the nitrogenase operon (*nifHDKENX*-*fdxB*-*nifQ*), two operons involved in nitrogenase maturation (*nifB*-*fdxN* and *iscN-nifUSVW*-*modD*), and the Rnf operon (*rnfABCDGEH*) encoding the Rnf electron transport complex. **(B)** Schematic representation of proteins involved in the nitrogen fixation and ammonia assimilation networks in *Z. mobilis*. Shown are the transmembrane Rnf complex (RnfABCDFE) thought to transfer electrons to nitrogenase via ferredoxin/flavodoxin, the nitrogenase complex (nifHDK), and the GS/GOGAT cycle of ammonia assimilation. Also depicted is the transcription factor NifA acting as a master regulator controlling the expression of nitrogen fixation genes and the putative role of regulatory protein P_*II*_ at regulating NifA activity. **(C)** Heatmap showing differential phosphorylation of nitrogen metabolism enzymes and regulatory proteins across growth conditions. The red (high) and green (low) color scale indicates relative changes (log_2_ fold-changes vs. anaerobic samples) in protein phosphorylation across growth conditions. Data represents the average of 5 biological replicates. For proteins that were not phosphorylated in the anaerobic control samples and in at least one more growth condition, fold-change comparisons were not made; instead, changes in phosphorylation status are shown as follows: *X* indicates no phosphorylation was detected, *P* indicates site is phosphorylated, and a dash (–) indicates that protein level was not quantitated due to its low abundance in the specified growth condition and phosphorylation was also not detected. For fold-change comparisons, the superscripts *a* and *b* indicate significant (adjusted *q* < 0.05, see section “Materials and Methods”) changes in phosphorylation under aerobic and N_2_-fixing conditions, respectively. Abbreviations: GS, Glutamine synthase; GOGAT, Glutamate synthase; NifH, Nitrogenase iron protein; NifD, Nitrogenase molybdenum iron protein; P_*II*_, Nitrogen regulatory protein; NifA, Nif specific regulatory protein; NifL, Nitrogen fixation regulatory protein; NifQ, Nitrogen fixation Q protein; RnfEGDC, Electron transport complex subunits.

Biological nitrogen fixation (i.e., reduction of atmospheric nitrogen to ammonia) in bacteria and archaea is carried out by the highly conserved nitrogenase complex. In most nitrogen-fixing bacteria, including *Z. mobilis*, the nitrogenase complex is encoded by the *NifHDK* genes. *NifHDK* expression is controlled by the transcription factor NifA, a master regulator that also controls expression of many other genes involved in nitrogen fixation (Curatti et al., 2005; Heiniger et al., 2012; Tsoy et al., 2016). In *Z. mobilis*, all *NifA*-regulated genes are located within a single chromosomal region (ZMO1808-37) that includes the *NifA* gene, the nitrogenase operon (*nifHDKENX*-*fdxB*-*nifQ*), two operons involved in nitrogenase maturation (*nifB-fdxN* and *iscN-nifUSVW-modD*), and the Rnf operon (*rnfABCDGEH*) encoding the Rnf electron transport complex (Figure 4A; Tsoy et al., 2016).

We observed substantial changes in phosphorylation of the nitrogenase subunits NifH (Fe protein, ZMO1823) and NifD (Fe-Mo protein α-subunit, ZMO1824) during N_2_-fixing growth. NifH was phosphorylated at Ser^90^ and NifD was phosphorylated at Ser^60^, Ser^53^, Thr^42^, and Ser^286^ exclusively under N_2_-fixing conditions (Figure 4C). NifD phosphorylation at Ser^2^ was observed in both anaerobic and N_2_-fixing conditions but displayed a significant increase in phosphorylation under N_2_-fixing conditions (Figure 4C). NifQ (ZMO1831), potentially involved in the incorporation of molybdenum into nitrogenase, was also phosphorylated (Ser^111^) only under N_2_-fixing conditions (Figure 4C).

In contrast to nitrogenase proteins, NifA (ZMO1816) was phosphorylated at Ser^200^ only during anaerobic growth (Figure 4C). In γ-proteobacteria, NifA activation is regulated via an interacting partner protein, NifL, whereas in α-proteobacteria and some β-proteobacteria that lack the *nifL* gene, activation of NifA is controlled by other regulatory mechanisms, mainly involving regulatory P_*II*_ proteins acting in response to the cellular nitrogen and carbon status (Jack et al., 2001; Dixon and Kahn, 2004; Martinez-argudo et al., 2004; Radchenko and Merrick, 2011). *Z. mobilis* lacks the *nifL* gene and the mechanism of NifA regulation remains unexplored. It is possible that dephosphorylation of NifA at Ser^200^ under aerobic and N_2_-fixing conditions, as well as the phosphorylation changes in the regulatory protein P_*II*_ described in the next section, may play a role on regulating the activity of this transcription factor in *Z. mobilis*.

Rnf proteins (RnfABCDGEH) form a transmembrane complex that catalyzes reverse electron flow from NADH to reduce ferredoxin/flavodoxin, which acts as the electron donor to nitrogenase (Curatti et al., 2005; Biegel et al., 2011; Bueno Batista and Dixon, 2019) (Figure 4B). We observed a significant decrease in phosphorylation of RnfD (Ser^178^) and RnfG (Thr^205^) proteins in N_2_-fixing conditions. In contrast, RnfC was phosphorylated (Ser^5^) exclusively under N_2_-fixing conditions (Figure 4C). Although the role of Rnf proteins has not been studied in *Z. mobilis*, changes in their phosphorylation status under N_2_-fixing conditions may affect their activity in transferring electrons to nitrogenase.

### Ammonia Assimilation (GS/GOGAT Cycle) and Regulatory Protein P_*II*_

Ammonia (NH_4_^+^) assimilation into glutamate takes places via the glutamine synthetase/glutamate synthase cycle (GS/GOGAT, ZMO0493, and ZMO1116). GS catalyzes the ATP-dependent production of glutamine from NH_4_^+^ and glutamate. GOGAT then takes this glutamine and α-ketoglutarate to produce two molecules of glutamate (Figure 4B). Glutamate may also be produced from NH_4_^+^ and α-ketoglutarate in a single reaction catalyzed by glutamate dehydrogenase (GDH), but this enzyme appears to be missing in *Z. mobilis*. GS activity is extensively regulated in response to intracellular levels of glutamine, α-ketoglutarate, and other metabolites. Post-translationally, GS is regulated via adenylyation/deadenylylation by the bifunctional adenylyl transferase (AT). AT activity is directly modulated by glutamine, which favors GS adenylyation (i.e., inactivation). AT activity is also regulated by regulatory protein P_*II*_: uridylyated P_*II*_ interacts with AT to promote GS deadenylylation (i.e., activation) while non-uridylyated P_*II*_ favors adenylyation. Uridylylation of P_*II*_ by uridylyl transferase (UT, ZMO0766) is in turn favored by low glutamine and high α-ketoglutarate levels (Figure 4B; Bueno Batista and Dixon, 2019).

We found that GS was phosphorylated at Ser^6^ and Ser^342^ and GOGAT was phosphorylated at Ser^376^ and Ser^380^ (Figure 4C). Under aerobic conditions, phosphorylation of GS and GOGAT increased significantly at all sites. Under N_2_-fixing conditions, only Ser^342^ in GS and Ser^380^ in GOGAT displayed significant increase in phosphorylation, although there was also a clear trend for increased phosphorylation of GOGAT at Ser^376^ (Figure 4C). Phosphorylation of GS/GOGAT enzymes may constitute a regulatory mechanism controlling activity of this ammonia assimilation cycle under aerobic and N_2_-fixing conditions.

In addition to GS/GOGAT enzymes, we also found regulatory protein P_*II*_ (ZMO0492) to be differentially phosphorylated at Tyr^51^ and Ser^26^ under different growth conditions (Figure 4C). Tyr^51^ phosphorylation increased significantly during N_2_-fixing conditions while phosphorylation on Ser^26^ was significantly higher in aerobic growth (Figure 4C). Thus, it is possible that these two P_*II*_ phosphorylation events may have unique, and potentially opposite, regulatory effects in N_2_-fixing vs. aerobic growth conditions in *Z. mobilis*.

### Ribosomal Proteins and Protein Biosynthesis

**FIGURE 5:**
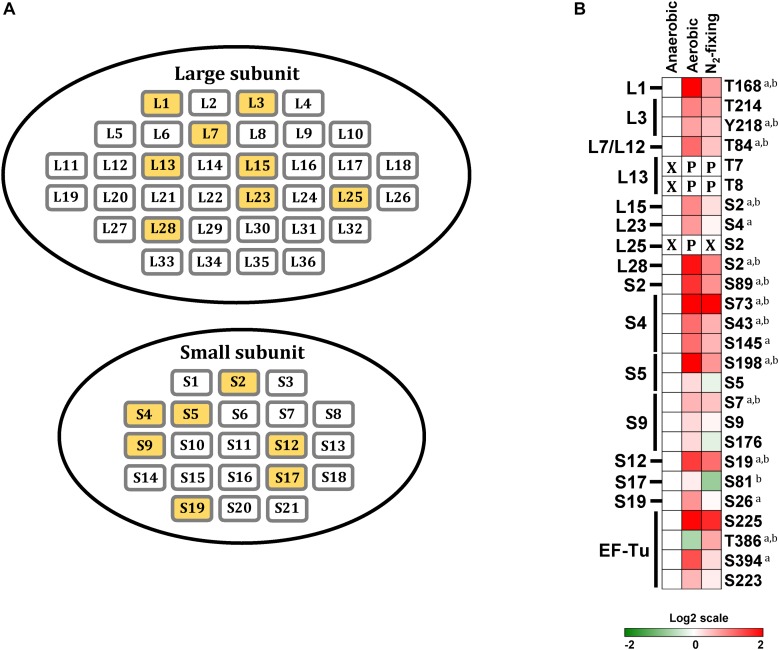
Phosphorylation of ribosomal proteins. **(A)** Ribosomal proteins of *Z. mobilis*. Yellow colored boxes indicate the phosphoproteins identified in this study. **(B)** Heatmap showing differential phosphorylation of ribosomal proteins and Elongation factor-Tu (EF-Tu) across growth conditions. The red (high) and green (low) color scale indicates relative changes (log_2_ fold-changes vs. anaerobic samples) in protein phosphorylation across growth conditions. Data represents the average of 5 biological replicates. For proteins that were not phosphorylated in the anaerobic control samples and in at least one more growth condition, fold-change comparisons were not made; instead, changes in phosphorylation status are shown as follows: *X* indicates no phosphorylation was detected, *P* indicates site is phosphorylated. For fold-change comparisons, the superscripts *a* and *b* indicate significant (adjusted *q* < 0.05, see section “Materials and Methods”) changes in phosphorylation under aerobic and N_2_-fixing conditions, respectively.

Ribosomal proteins, together with rRNA, make up the small and large ribosomal subunits, termed 30S and 50S in bacteria, responsible for cellular protein synthesis. We found that 15 out of 57 ribosomal proteins were phosphorylated in at least one growth condition. Within the large subunit, L1, L3, L13, L15, L23, L25, L28, and L7/L12 stalk complex were phosphorylated; within the small subunit, S2, S4, S5, S9, S12, S17, and S19 were phosphorylated (Figure 5A). Phosphorylation of ribosomal proteins has been observed in several bacteria, including *L. lactis*, *B. subtilis*, *M. tuberculosis*, *E. coli*, and *R. palustris* (Macek et al., 2007b; Soufi et al., 2008; Aivaliotis et al., 2009; Ravichandran et al., 2009; Soung et al., 2009; Parker et al., 2010; Manteca et al., 2011; Hu et al., 2012; Lin et al., 2015). All of the ribosomal proteins that we found phosphorylated in *Z. mobilis*, except for L15, have also been reported to be phosphorylated in *E.coli* (Macek et al., 2007a, b; Soufi et al., 2008; Prisic et al., 2010; Hu et al., 2012; Lin et al., 2015). Interestingly, we observed a clear trend of increased phosphorylation in ribosomal proteins during aerobic and N_2_-fixing growth. 12 out of 15 phosphorylated ribosomal proteins displayed increased phosphorylation on single or multiple sites under aerobic and N_2_-fixing growth conditions (Figure 5B).

The elongation factor EF-Tu catalyzes binding of aminoacyl-tRNAs (aa-tRNA) to the ribosome (Sajid et al., 2011). EF-Tu forms a ternary complex with GTP via its N-terminal domain (domain I) and aminoacyl-tRNA (aa-tRNA) through its β-barrel domains (domain II and III) (Nissen et al., 1995). EF-Tu phosphorylation at Thr^382^ in *E.coli*, Thr^63^ in *B. subtilis*, and Thr^118^ in *M. tuberculosis*, has been shown to block protein synthesis by interfering with GTP binding (Sajid et al., 2011; Pereira et al., 2015; Talavera et al., 2018). Here we observed that EF-Tu phosphorylation at Thr^386^, equivalent to Thr^382^ in *E. coli*, decreased significantly during aerobic growth but increased under N_2_-fixing conditions (Figure 5B), suggesting that *Z. mobilis* might also regulate protein synthesis via EF-Tu phosphorylation. Additionally, we found three other phosphorylation sites (Ser^223^, Ser^225^, and Ser^394^) on EF-Tu that were located on domains II and III. These phosphorylation events could potentially modulate EF-Tu interaction with aa-tRNAs (Nissen et al., 1995). These three sites displayed a trend of increased phosphorylation under both aerobic and N_2_-fixing conditions (Figure 5B). Interestingly, we also found multiple phosphorylation sites on several aa-tRNA ligases that displayed differential phosphorylation under different growth conditions, but the potential significance of this is unclear (Figure 2).

The L7/L12 stalk complex is known to physically interact with elongation factors EF-Tu and EF-G to promote ternary complex binding to the ribosome (Agrawal et al., 1999, 2002; Ban et al., 2000; Kothe et al., 2004; Savelsbergh et al., 2005). Lys^84^ of L7/L12 is a highly conserved surface residue that is thought to be critical for its interaction with EF-Tu; its mutation to alanine has been reported to significantly decrease binding efficiency of the ternary complex to the ribosome (Kothe et al., 2004). Interestingly, Lys^84^ is replaced with threonine in *Z. mobilis*. We found that phosphorylation of this Thr^84^ residue increased significantly under aerobic and N_2_-fixing conditions (Figure 5B), which could potentially affect the interaction of L7/L12 with EF-Tu and thereby ternary complex binding to the ribosome.

L3 and L13 are part of a protein cluster in the large ribosomal subunit that provides important interaction sites for elongation factors (Ban et al., 2000). We found that L13 was phosphorylated (Thr^7^ and Thr^8^) exclusively under aerobic and N_2_-fixing conditions while L3 (Thr^214^ and Tyr^218^) phosphorylation increased under these two growth conditions (Figure 5B), which may potentially affect the interaction with elongation factors and influence ribosomal function.

## Discussion

This study represents the first genome wide phosphoproteome analysis of *Z. mobilis*. We identified 125 unique phosphorylated proteins belonging to metabolic pathways and cellular processes such as glycolysis, TCA cycle, protein biosynthesis, electron transport, nitrogen fixation, and ammonia assimilation (Figure 1). Quantitative analysis revealed widespread changes in protein phosphorylation across anaerobic, aerobic, and N_2_-fixing growth conditions (Figure 2).

Phosphorylation of glycolytic enzymes appears to be prevalent in bacteria, highlighting its potential as a conserved regulatory mechanism of this pathway (Macek et al., 2007a, b; Soufi et al., 2008; Manteca et al., 2011; Lin et al., 2015). For example, the activity of pyruvate phosphate dikinase (PPDK), an enzyme involved in glycolysis/gluconeogenesis and CO_2_ assimilation, is positively regulated by phosphorylation (Thr^487^) in the phototrophic bacterium *R. palustris* (Hu et al., 2012). Also, the activity of the glycolytic enzyme enolase has been shown to decrease by phosphorylation of Ser^336^, Thr^363^, and Ser^367^ in the pathogen *Bacillus anthracis* (Virmani et al., 2019). In *Z. mobilis*, we observed a generalized increase, compared to baseline anaerobic growth, in the phosphorylation of most glycolytic enzymes during aerobic and N_2_-fixing growth conditions (Figure 3). The specific rate of glucose consumption changes substantially in these two growth conditions (Kremer et al., 2015; Strazdina et al., 2018), and it is possible that some of the phosphorylation events that we identified may regulate glycolytic enzyme activity leading to overall changes in flux.

We observed substantial changes in the phosphorylation status of enzymes and regulatory proteins involved in nitrogen fixation and ammonia assimilation, including nitrogenase, the Rnf electron transport complex, the transcription factor NifA, GS-GOGAT cycle enzymes, and the P_*II*_ regulatory protein (Figure 4). These observations suggest that protein phosphorylation may play a critical role at regulating all aspects of nitrogen metabolism in *Z. mobilis*. For example, increased phosphorylation of the nitrogenase complex during N_2_-fixation suggests that these phosphorylation events may promote its activation. There are several known mechanisms that regulate the activity of the nitrogenase complex, including regulation by P_*II*_ and ADP-ribosylation (Huergo et al., 2012, 2013; Sarkar et al., 2012; Moure et al., 2013). However, to the best of our knowledge, changes in the phosphorylation status of nitrogenase have never been reported in any bacteria. Similarly, we have not come across any reported example of NifA phosphorylation. The activity of the GS-GOGAT cycle is also extensively regulated through various mechanisms, including allosteric regulation and PTM; our results suggests that in *Z. mobilis*, GS and GOGAT may also be regulated by phosphorylation.

In proteobacteria, P_*II*_ signal transduction proteins are known to regulate both nitrogen fixation and nitrogen assimilation. P_*II*_ activity is allosterically regulated by intracellular glutamine, ADP, ATP, and a-ketoglutarate levels as well as by post-translational modification of its flexible T-loop region, which induces conformational changes that modulate its ability to interact with target proteins (Huergo et al., 2013). Specifically, the highly conserved T-loop residue Tyr^51^ may be modified in response to changes in cellular nitrogen status by uridylation (*E. coli* and other bacteria), adenylylation (*S. coelicolor*, *Corynebacterium glutamicum*) or nitration (*anabaena*) (Forchhammer and De Marsac, 1994; Kloft and Forchhammer, 2005; Huergo et al., 2013). In addition, phosphorylation of T-loop residue Ser^49^ under N_2__–_fixing conditions or in response to high AKG concentrations has been observed in cyanobacteria (*S. elongatus* and *Synechocystis* sp.) (Forchhammer and De Marsac, 1994, 1995; Kloft and Forchhammer, 2005). In *Z. mobilis*, we observed differential phosphorylation of P_*II*_ at Tyr^51^ across growth conditions (Figure 4C). Phosphorylation of P_*II*_ at Tyr^51^ has not been reported in any bacteria, but it is possible that *Z. mobilis* utilizes this phosphorylation instead of other PTMs to regulate P_*II*_ activity and nitrogen metabolism. Similarly, although outside of the T-loop, differential P_*II*_ phosphorylation at Ser^26^ could also play a role in regulating nitrogen metabolism. Finally, it has been reported that P_*II*_ can bind to RnfC in the Rnf complex to inactivate its activity (Sarkar et al., 2012) It would be interesting to investigate whether the observed changes in P_*II*_ or RnfC phosphorylation in *Z. mobilis* modulate Rnf complex inhibition by P_*II*_ and disrupt electron flow to the nitrogenase complex.

Paralleling observations in other bacteria (Aivaliotis et al., 2009; Soung et al., 2009; Mikulík et al., 2011; Lin et al., 2015), we found that a significant fraction of ribosomal proteins (24%) are phosphorylated in *Z. mobilis* (Figure 5). *In vitro* experiments have shown that phosphorylation of ribosomal proteins can alter ribosomal activity (Traugh and Traut, 1972; Mikulík et al., 2001, 2011). Therefore, it is possible that the generalized increase in phosphorylation of ribosomal proteins during aerobic and N_2_-fixing growth may play a role at regulating translation in *Z. mobilis* under these conditions. For example, changes in phosphorylation of L7/L12, L3, and L13 could potentially affect ribosomal activity by modulating interactions with elongation factors EF-Tu and EF-G. Similarly, differential phosphorylation of EF-Tu across growth conditions may also serve as a regulatory mechanism of protein synthesis (Figure 5B).

Our results show that overall protein phosphorylation in *Z. mobilis* increases under what may be considered “non-optimal” growth conditions, i.e., aerobic and N_2_-fixing conditions vs. anaerobic growth with abundant ammonia. This suggests that phosphorylation may be particularly important for regulating metabolism during adverse growth conditions.

## Conclusion

The genome-wide phosphoproteome analysis of *Z. mobilis* that we have presented here provides new knowledge regarding the specific proteins, pathways, and cellular processes that may be regulated by phosphorylation in this important industrial organism. The results of this study provide a useful road map and a unique hypothesis-generating resource to establish future genetic and biochemical experiments that investigate the physiological role of specific phosphorylation events in *Z. mobilis*.

## Data Availability

The mass spectrometry proteomics data have been deposited to the ProteomeXchange Consortium via the PRIDE partner repository with the dataset identifier PXD014065 and can be accessed via this link: http://www.ebi.ac.uk/pride/archive/projects/PXD014065.

## Author Contributions

MT, AH, JC, and DA-N designed the study. MT prepared the samples for the analysis. AH performed the mass spectrometry experiments. MT, AH, and DA-N analyzed the data. MT and DA-N wrote the manuscript. All authors read and approved the final version of the manuscript.

## Conflict of Interest Statement

The authors declare that the research was conducted in the absence of any commercial or financial relationships that could be construed as a potential conflict of interest.
